# Mice with early retinal degeneration show differences in neuropeptide expression in the suprachiasmatic nucleus

**DOI:** 10.1186/1744-9081-6-36

**Published:** 2010-07-06

**Authors:** Linda Ruggiero, Charles N Allen, R Lane Brown, David W Robinson

**Affiliations:** 1 Center for Research on Occupational and Environmental Toxicology, Oregon Health & Science University, (3181 SW Sam Jackson Park Road), Portland, (97239) USA; 2 Neuroscience Graduate Program, Oregon Health & Science University, (3181 SW Sam Jackson Park Road), Portland, (97239) USA; 3 Department of Biology, Fordham University, (441 E Fordham Rd), Bronx, (14058) USA; 4 Department of Veterinary & Comparative Anatomy, Pharmacology, and Physiology, Washington State University, (205 Wegner Hall), Pullman, (99164), USA

## Abstract

**Background:**

In mammals, the brain clock responsible for generating circadian rhythms is located in the suprachiasmatic nucleus (SCN) of the hypothalamus. Light entrainment of the clock occurs through intrinsically photosensitive retinal ganglion cells (ipRGCs) whose axons project to the SCN via the retinohypothalamic tract. Although ipRGCs are sufficient for photoentrainment, rod and cone photoreceptors also contribute. Adult CBA/J mice, which exhibit loss of rod and cone photoreceptors during early postnatal development, have greater numbers of ipRGCs compared to CBA/N control mice. A greater number of photosensitive cells might argue for enhanced light responses, however, these mice exhibit attenuated phase shifting behaviors. To reconcile these findings, we looked for potential differences in SCN neurons of CBA/J mice that might underly the altered circadian behaviors. We hypothesized that CBA/J mice have differences in the expression of neuropeptides in the SCN, where ipRGCs synapse. The neuropeptides vasoactive intestinal peptide (VIP) and vasopressin (VP) are expressed by many SCN neurons and play an important role in the generation of circadian rhythms and photic entrainment.

**Methods:**

Using immunohistochemistry, we looked for differences in the expression of VIP and VP in the SCN of CBA/J mice, and using a light-induced FOS assay, we also examined the degree of retinal innervation of the SCN by ipRGCs.

**Results:**

Our data demonstrate greater numbers of VIP-and VP-positive cells in the SCN of CBA/J mice and a greater degree of light-induced FOS expression.

**Conclusions:**

These results implicate changes in neuropeptide expression in the SCN which may underlie the altered circadian responses to light in these animals.

## Background

In mammals, retinal input to the brain clock, located in the suprachiasmatic nucleus (SCN) of the hypothalamus, is required for entrainment of circadian rhythms. Photoreception within the retina is accomplished by rod and cone photoreceptors and intrinsically photosensitive retinal ganglion cells (ipRGCs), which express the photopigment, melanopsin [1-3]. Rods and cones are not required for entrainment. However, they do play a role since melanopsin knock-out mice retain non-image forming (NIF) responses to light, such as photoentrainment and the pupillary light reflex (PLR), that are eliminated in mice lacking rods, cones and melanopsin [4,5].

We have previously shown that CBA/J mice, which exhibit an early loss of outer retinal photoreceptors, have greater numbers of ipRGCs compared to CBA/N control mice [6]. A greater number of photosensitive cells might argue for enhanced light responses, however, these mice exhibit attenuated phase shifting [6,7]. Studies that assay the PLR in CBA/J mice suggest that the melanopsin pathway is functional at the level of the retina [6]. We hypothesize, therefore, that differences in circadian behaviors are due to changes in central processing.

SCN neurons express a variety of neuropeptides, with γ-Aminobutyric acid (GABA) as the most prominent [8-10]. In the mouse SCN, a set of neurons expressing vasoactive intestinal peptide (VIP) are located in the ventral-medial SCN, while the dorsal-lateral SCN is populated by cells expressing vasopressin (VP) [11]. VIP-expressing cells receive retinal input. In these cells, expression of the clock gene, *Per1*, increases in response to light in a manner that correlates with behavioral phase shifting responses [12,13]. Cells expressing VIP are thought to relay photic information to the VP-containing cells, because application of VIP induces phase-shifts in the circadian expression of VP in a similar manner to light [14]. In addition, VIP knockout mice display behavioral arrythmicity as well as desynchronization of the clock with the environment [15,16].

We examined the expression of VIP-and VP-containing cells and retinal innervation in CBA/J and CBA/N mice. Our data demonstrate greater numbers of VIP-and VP-positive cells and greater FOS expression in response to light and in CBA/J mice, which may underlie altered central processing of photic signals.

## Materials and methods

### Animals

Adult male CBA/J mice (Jackson Laboratory, Bar Harbor, ME, USA), which carry the *Pde6b^rd1 ^*mutation and exhibit blindness by weaning age, were used to examine the effects of early retinal degeneration on circadian function and retinal innervation of the SCN. For controls, we used male CBA/N mice (National Cancer Institute, Frederick, MD, USA), which have the same genetic background as the CBA/J mice, but lack the phosphodiesterase mutation and are, therefore, visually intact. The mice were housed in facilities that permit the maintenance of a 12-hour light-dark (LD 12:12) cycle [6]. All procedures were carried out in compliance with the guidelines of the National Institutes of Health. The Institutional Animal Care and Use Committee of Oregon Health & Science University approved all protocols in advance.

### Tissue preparation

Animals were deeply anesthetized with isoflurane and perfused intracardially with 4% paraformaldehyde (PFA) (pH 7.4) for 10 min at zeitgeber time (ZT) 6. The brains were removed and postfixed in PFA overnight at 4°C. Following fixation, the brains were cryoprotected by successive immersion in phosphate-buffered solutions containing 10% and 30% sucrose at 4°C overnight. The tissue was embedded in Thermo Shandon Cryochrome (Thermo Scientific, Pittsburgh, PA, USA), fast-frozen over dry-ice mixed with 100% ethanol for 3-5 minutes and stored at -80°C.

### Antibodies

Rabbit polyclonal antibodies directed against VIP (Peninsula Laboratories, San Carlos, CA, USA) or VP (Abcam, Cambridge, MA, USA) were diluted (1:500) in blocking solution (1% BSA + 0.3% Triton-X). The rabbit polyclonal FOS antibody (Oncogene Science, Manhasset, NY, USA) was diluted (1:250) in blocking solution. Alexa-488 labeled goat anti-rabbit IgG (Molecular Probes, Eugene, OR, USA) diluted in blocking solution (1:1000) was used as a secondary antibody.

### Immunohistochemistry

Embedded brains were sectioned (20 μm) on a Leica 1720 digital cryostat. Sections containing SCN were rinsed in 0.1 M PB with 0.3% Triton-X for 15 min and placed in blocking solution for 1 hour at RT. For VIP and VP studies, the tissue was incubated in primary antibody for 48 hours at 4°C. For the light-induced FOS expression experiments, the tissue was incubated in primary antibody for 72 hours at 4°C. Sections were rinsed with 0.1 M PB with 0.3% Triton-X and incubated in secondary antibody for 2 hours at RT. The tissue was rinsed with 0.1 M PB, counterstained with the nuclear stain, DAPI (80 ng/ml) for 1-3 min at RT and mounted on a glass slide in Aqua Mount (Fisher Scientific, Pittsburgh, PA, USA). Immunostained tissue was imaged by fluorescence microscopy at 20 × using a Zeiss Axioscope 2 TM.

### Light-induced *FOS *expression

CBA/J and CBA/N mice were maintained in constant darkness (DD) for at least 10 days, and the free-running periods were calculated using ClockLab (Actimetrics Software, Wilmette, IL, USA). At circadian time (CT) 16, the mice were exposed to a white light pulse administered by a 32-W fluorescent bulb (500 lux) for 1 hour then returned to DD for 90 min. Mice were anesthetized with isoflurane and perfused under dim red light. Brains were removed and processed as described above. Control mice were kept in the same lighting conditions without the presentation of a light pulse and sacrificed at the same CT. The number of FOS positive cells was counted, and the percent increase in FOS in response to light was calculated by dividing the average number of cells in mice exposed to light by the average number of cells in mice in DD.

### Cell counts

VIP, VP and FOS staining were examined in CBA/J and CBA/N mice. Brain sections were collected and stained as described above. The average number of VIP-and VP-positive cell bodies was calculated in each section and averaged among three 20 μm coronal sections along the middle of the rostral-caudal axis and separated by 40-60 μm. Cell counts were performed using ImageJ software [17]. DAPI staining was used to ensure that the level of coronal brain sections were comparable across the tissue samples examined from each animal. Image contrast was adjusted using Axiovision software (Carl Zeiss Microimaging, Germany).

### Statistics

Averages are reported as the mean ± SD. Significance was determined using the Student's t-Test.

## Results

### CBA/J mice have greater numbers of VIP-and VP-positive cells

**Figure 1:**
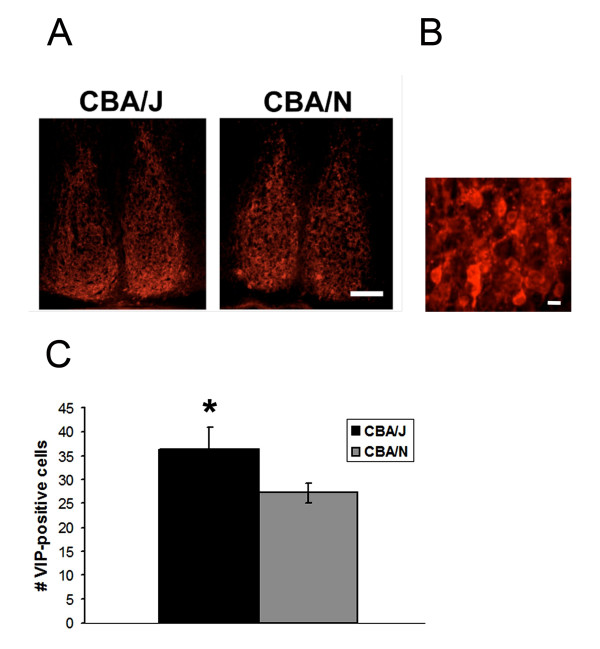
**VIP-positive cells in the SCN of CBA/J and CBA/N mice**. A. Coronal SCN sections stained with an antibody against VIP show that in both CBA/J and CBA/N mice, VIP-positive cells bodies are found primarily in the ventral SCN, with fibers projecting dorsally. B. Magnified area indicating the ability to count cell bodies stained for VIP. Scale bar = 20 μm. C. The average number of VIP-positive cells detected per SCN is greater in the SCN of CBA/J mice compared to CBA/N. Scale bar = 100 μm. * = p = 0.04, t_10 _= -2.4.

VIP-containing cells are found in the ventral portion of the mouse SCN and receive retinal input. To examine whether early retinal degeneration altered neuropeptide expression in the SCN, we examined the number of VIP-positive cells in CBA/J (n = 7) and CBA/N (n = 5) mice. Figure 1A shows that in both CBA/J and CBA/N mice, VIP-positive cell bodies were concentrated in the ventral SCN with few cells in the dorso-medial SCN, and VIP-positive fibers projected dorsally. Upon counting the cells (Figure 1B), we found that there were significantly greater numbers of detectable VIP-positive cells in the CBA/J mice compared to CBA/N mice (CBA/J = 37 ± 8 cells; CBA/N = 27 ± 5 cells, p = 0.04; t_10 _= -2.4) (Figure 1C).

**Figure 2:**
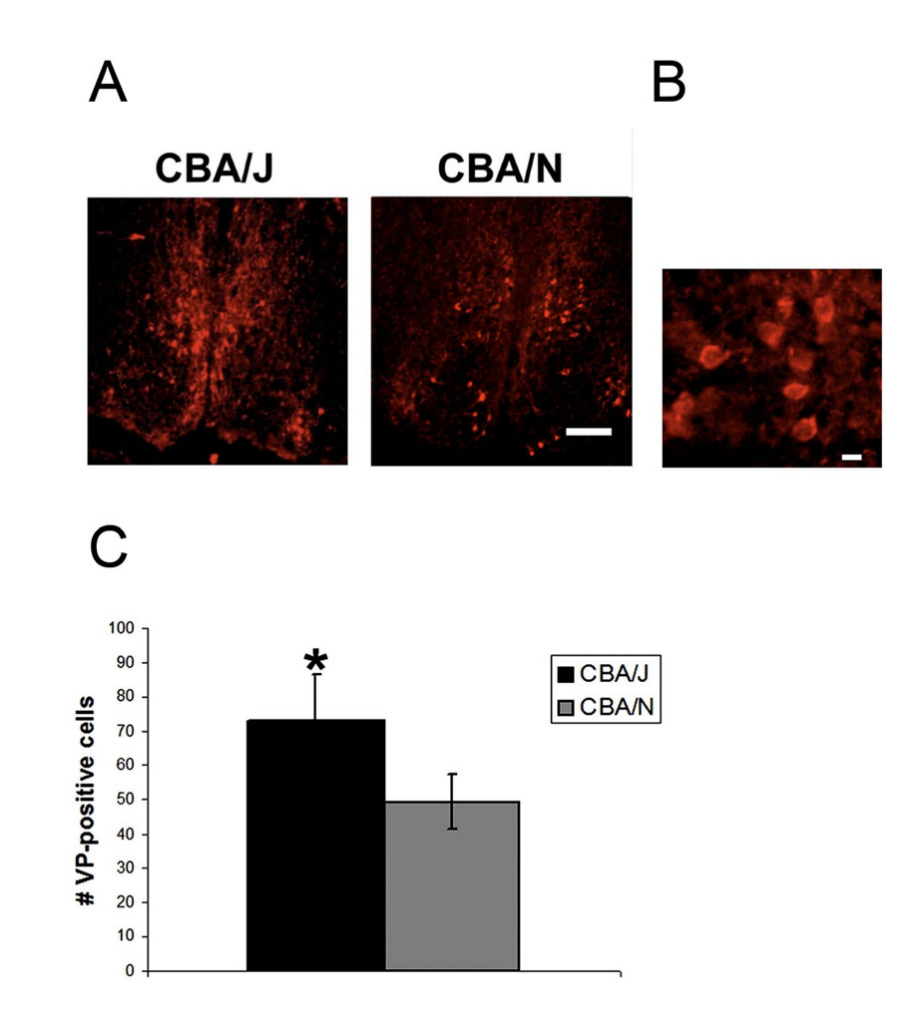
**VP-positive cells in the SCN of CBA/J and CBA/N mice**. A. Coronal SCN sections stained with an antibody against VP show that in both CBA/J and CBA/N mice, VP-positive cell bodies are found primarily in the dorsal shell. B. Magnified area indicating the ability to count cell bodies stained for VP. Scale bar = 20 μm. C. The average number of detectable VP-positive cells per SCN is greater in CBA/J mice compared to CBA/N. Scale bar = 100 μm. * = p = 0.01, t_10 _= 3.15.

To examine differences in SCN output neurons of CBA/J mice, coronal brain sections from CBA/J (n = 8) and CBA/N (n = 4) mice were stained with an antibody against VP. In both strains, VP-positive cells were found dorsally in the SCN. For the most part, cell bodies were not observed in the ventral SCN (Figure 2A). Significantly greater numbers of detectable cells were present in CBA/J mice compared to controls (CBA/J = 73 ± 14 cells; CBA/N = 49 ± 8 cells, p = 0.01, t_10 _= 3.15) (Figure 2B, C).

### Light induction of *FOS *expression is greater in CBA/J mice

**Figure 3:**
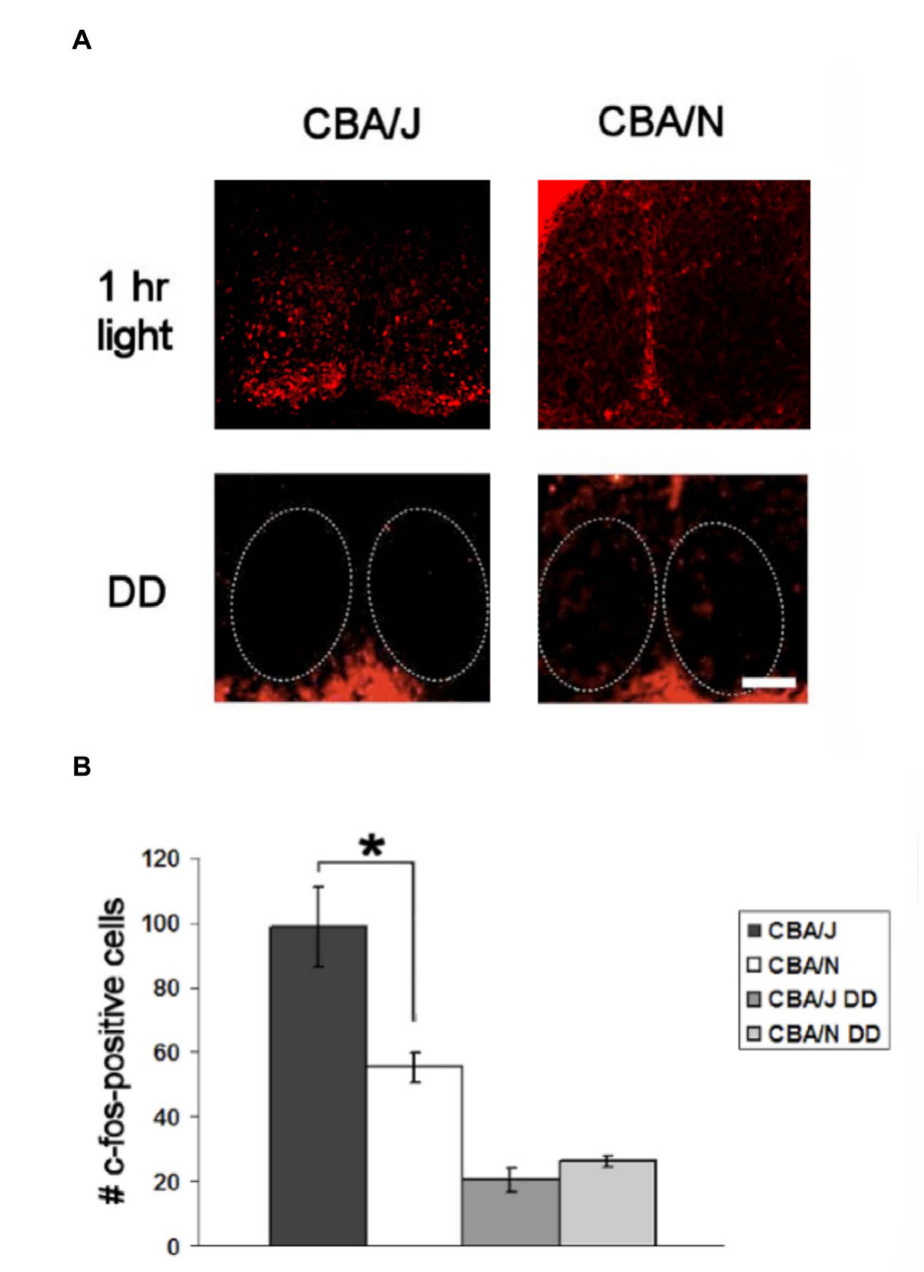
**Light-induced FOS expression in the SCN of CBA/J and CBA/N mice**. A. There are greater numbers of FOS-positive cells in the SCN in response to a 1hr light pulse is greater in CBA/J (dark grey) than CBA/N mice (white). Light grey bars show that FOS expression is significantly less in animals kept in DD and does not differ between strains (light grey bars). * = p = 8.9 × 10^-5^, t_8 _= 7.2.

We examined light-induced FOS expression in the SCN of CBA/J and CBA/N as an indication of functional retinal innervation. CBA/J (n = 5) and CBA/N (n= 5) mice kept in DD were exposed to a pulse of bright light for 1 hour at CT 16. In response to light, FOS expression was induced in the SCN of both strains. The gain of the fluorescent signal was increased equally in all sections in order to better visualize the staining. This caused some cells to appear brighter than others, which does not represent a change in the level of protein expression. Though concentrated in the ventral region, staining was present throughout the SCN, consistent with previously reported work [18-20]. The percent increase in FOS positive cells in mice exposed to light compared DD was significantly greater in CBA/J mice compared to CBA/N (CBA/J, 4.8-fold increase; CBA/N, 2.1-fold increase) (CBA/J, 99 ± 13 cells; CBA/N, 56 ± 5, p = 8.9 × 10^-5^, t_8 _= 7.2). Expression was low in mice kept in DD (CBA/J, n = 3; CBA/N, n = 3) with no exposure to light, suggesting that FOS induction was the result of light exposure. These values were not statistically different from each other (CBA/J, 20. ± 4 cells; CBA/N, 26 ± 2. cells, p = 0.07, t_4 _= -2.4) (Figure 3).

## Discussion

CBA/J mice have attenuated phase-shifting responses to light compared to CBA/N controls [6,7]. These results are surprising because CBA/J mice have greater numbers of ipRGCs [6]. A reasonable hypothesis is that the increased number of ipRGCs would result in enhanced behavioral responses to light. The work described here attempts to reconcile these findings by examining changes in central processing that could explain the differences in behavior.

### VIP-expressing cells

To identify the relationship between the increased numbers of ipRGCs and the attenuated behaviors in CBA/J mice, we examined potential changes in neuropeptide expression in the SCN. VIP is expressed by many SCN neurons and is often used as a marker for the ventral SCN. Mice lacking VIP or its receptor, VPAC2, have weak circadian rhythms in DD, while over-expressing the VPAC2 receptors shortens the free-running period in DD [21,22]. Genetic deletion of VIP or the VPAC2 receptor disrupts rhythmic clock gene expression, the rhythmic action potential firing of SCN neurons, and behavioral circadian rhythms [23]. Synchrony of individual oscillators and behavioral rhythmicity can be rescued by application of VIP or the VPAC2 receptor agonist RO 25-1553 [24,25]. In addition, VIP is known to play a role in phase-shifting because injection of VIP into hamster SCN causes phase-shifts in locomotor activity that are similar to those produced by light [26,27]. Together these data suggest an important role for VIP in light transmission and coordination in the SCN and in circadian behaviors.

In these experiments, we sought to determine whether there are differences in VIP expression in SCN neurons that might account for the changes of photic entrainment. We found a greater number of VIP-positive cells in the SCN of CBA/J mice. This finding was unexpected because it suggests that greater numbers of light responsive cells are associated with attenuated circadian behaviors. This finding mirrors that of our previous work, which showed that CBA/J mice have greater numbers of light responsive cells in the retina compared to controls [6].

### VP-expressing cells

To further explore changes in the SCN, we looked at neurons that function to output circadian timing signals, which might be influenced by VIP. VP is expressed in neurons of the dorsal-lateral SCN and is released in a circadian manner and relies on input from VIP-expressing cells for synchronization, implicating its role in circadian output. VIP also induces phase shifts in VP expression, and mice with a null mutation in the gene for the VIP receptor, VPAC2, do not express rhythms in VP expression [22,27]. We now report that CBA/J mice have greater numbers of detectable cells expressing VP compared to controls. This finding shows that in addition to greater numbers of input cells, CBA/J mice also have greater numbers of neurons involved in output pathways.

### Retinal innervation

Retinal input to the SCN is necessary for light entrainment; therefore changes in retinal innervation could explain differences in circadian behavior. We measured light-induced FOS expression in the SCN of the CBA/J mice at a time when a light pulse was shown to affect an attenuated phase-shift in circadian behavior. Given that our previous work demonstrates that outer retinal degeneration during development results in greater numbers of ipRGCs, and that there are greater numbers of VIP-positive cells in the SCN, we hypothesized that there would be a greater degree of retinal innervation in CBA/J mice compared to controls. We found that there were greater numbers of FOS positive cells in CBA/J mice compared to controls. In both strains, the FOS positive cells were located throughout the SCN, which is in agreement with previous studies [18-20]. These data suggest that there are greater numbers of light sensitive cells in the SCN of CBA/J mice. Whether FOS is involved in the entrainment pathway or if it is simply a marker for an early response to light is unclear. However, a change in the number of cells expressing FOS in response to light would suggest differences in functional innervation of the SCN, which could underlie changes in central processing. These data support the conclusion that the alterations in retinal and SCN anatomy observed in CBA/J mice also reflect a functional change.

In attempting to reconcile the observations that CBA/J mice have greater numbers of cells involved in circadian entrainment, but have attenuated phase changes, it is important to note that the SCN is a heterogeneous structure made up of many neurons expressing different peptides. One molecule of great importance is γ-aminobutyric acid (GABA), which is expressed in most SCN neurons and plays a major role in neurotransmission. The exact mechanism by which GABAergic synaptic transmission translates into a behavior is unclear, however, GABA can synchronize and phase-shift clock cells [28]. In hamsters, injection of the GABA receptor agonist, baclofen, into the SCN decreases the animals' phase-shifting responses to light in wheel running paradigms [29]. This suggests that activation of GABA receptors plays a role in attenuating behavioral responses to light. In mouse SCN, GABA is expressed within approximately 70% of VIP-expressing cells [30]. VIP enhances inhibitory transmission by increasing the frequency of IPSCs mediated by GABA in the SCN [31]. As a result, the increase in VIP-positive cell numbers in CBA/J mice might lead to an increased modulation of GABAergic activity.

VP is also colocalized with GABA [32]. It is possible that the increase in VP cells could play a role in the behaviors of CBA/J mice if the effects are mediated by an increase in GABA levels. Further work is needed to better understand how communication among VIP, VP, GABA and other neuropeptides within the SCN influences circadian function.

Retinal innervation is an important part of establishing proper pathways during development. The number of cells and connections they make are dependent on intrinsic properties of the cells and the target being innervated. The loss of target tissues can lead to an increase in cell death of the innervating cells, suggesting that the cell depends on the target for survival cues. Experimentally increasing the number of targets decreases the degree of developmental cell death [33,34]. In addition, the reduction of afferent input to the target results in a significant increase in neuronal death. This has been shown in the retina and superior cervical ganglion of the rat [34-36]. Together these data suggest that retinal innervation plays a role in determining cell number of the target cell as well as the neuron reaching it.

If developmental changes in retinal innervation affect the development of the SCN, this could explain why CBA/J mice have attenuated phase-shifting responses, while other animal models of retinal degenration, such as the *rd/rd *mouse and RCS rat do not [37,38]. These animals lose photoreceptors later in development, after the retina has matured, unlike the CBA/J mice, which lose outer retinal layers during postnatal development. Changes in the degree of innervation during development could explain the differences in ipRGC number as well as differences in the SCN.

## Limitations

The data presented here suggest that loss of photoreceptors during early postnatal development impacts circadian system function. Our light-induced FOS data suggest that there is a change in functional innervation of the SCN in CBA/J mice because there are greater numbers of FOS-expressing cells in response to light. This would be expected because there are greater numbers of ipRGCs. However, it would be helpful to have anatomical data to better correlate the findings of increased ipRGCs in the retina with changes in neuropeptide expression the SCN. We have performed intraocular injections with labeled cholera toxin in order to trace RGC axons and label their terminals in the SCN. This method was limited, however, as the fibers of RGCs are extremely dense, and it was difficult to resolve synapse locations using the techniques available to us. Having a method to specifically label ipRGC terminals would have enabled us to determine whether the greater numbers of ipRGCs terminate on the VIP-containing cells, which were also found in greater numbers in CBA/J mice. Such data would provide further evidence to link changes in the developing retina with changes in the SCN.

In addition, because the SCN is composed of a heterogeneous population of neurons, which express diverse neuropeptides, it is difficult to sort out the potential contributions of these various pathways to altered circadian behaviors.

## Conclusions

Our data demonstrate that the retinal degeneration during development seen in the CBA/J mice impacts the central processing responsible for circadian behaviors. It suggests a role for maturation of rods and cones in influencing circadian function. However, it is unclear whether degeneration in the retina is responsible for changes in the SCN. A greater number of light responsive cells could translate into attenuated circadian behaviors if there is a sign inversion within central processing. This could occur through enhanced inhibitory synaptic transmission, mediated by GABA. Further work would need to look at the contributions of additional signaling pathways both in the mature animal and during development.

## Abbreviations

CT: circadian time; DD: constant darkness; GABA: γ-Aminobutyric acid; ipRGCs: intrinsically photosensitive retinal ganglion cells; LD: light/dark; NIF: non-image forming; PFA: paraformaldehyde; PLR: pupillary light reflex; SCN: suprachiasmatic nucleus; VIP: vasoactive intestinal peptide; VP: vasopressin; ZT: zeitgeber time.

## Competing interests

The authors declare that they have no competing interests.

## Authors' contributions

LR did all of the experiments. LR, RLB, CNA and DR designed the experiments and wrote the manuscript. All authors read and approved the final manuscript.

## References

[B1] Berson DM Dunn FA Takao M Phototransduction by retinal ganglion cells that set the circadian clock Science 2002 295 1070 1073 10.1126/science.1067262 11834835

[B2] Hattar S Liao HW Takao M Berson DM Yau KW Melanopsin-containing retinal ganglion cells: architecture, projections, and intrinsic photosensitivity Science 2002 295 1065 1070 10.1126/science.1069609 11834834 PMC2885915

[B3] Panda S Sato TK Castrucci AM Rollag MD DeGrip WJ Hogenesch JB Provencio I Kay SA Melanopsin (Opn4) requirement for normal light-induced circadian phase shifting Science 2002 298 2213 2216 10.1126/science.1076848 12481141

[B4] Hattar S Lucas RJ Mrosovsky N Thompson S Douglas RH Hankins MW Lem J Biel M Hofmann F Foster RG Yau KW Melanopsin and rod-cone photoreceptive systems account for all major accessory visual functions in mice Nature 2003 424 76 81 10.1038/nature01761 12808468 PMC2885907

[B5] Lucas RJ Hattar S Takao M Berson DM Foster RG Yau KW Diminished pupillary light reflex at high irradiances in melanopsin-knockout mice Science 2003 299 245 247 10.1126/science.1077293 12522249

[B6] Ruggiero L Allen CN Brown RL Robinson D The development of melanopsin-containing retinal ganglion cells in mice with early retinal degeneration Eur J Neurosci 2009 2 359 67 10.1111/j.1460-9568.2008.06589.x PMC2764118 19200239

[B7] Yoshimura T Nishio M Goto M Ebihara S Differences in circadian photosensitivity between retinally degenerate CBA/J mice (rd/rd) and normal CBA/N mice (+/+) J Biol Rhythms 1994 9 51 60 10.1177/074873049400900105 7949306

[B8] Moore RY Entrainment pathways and the functional organization of the circadian system Prog Brain Res 1996 111 103 119 full_text 8990910 10.1016/s0079-6123(08)60403-3

[B9] Hamada T LeSauter J Venuti JM Silver R Expression of Period genes: rhythmic and nonrhythmic compartments of the suprachiasmatic nucleus pacemaker J Neurosci 2001 21 7742 7750 11567064 10.1523/JNEUROSCI.21-19-07742.2001 PMC3275352

[B10] Morin LP SCN organization reconsidered J Biol Rhythms 2007 22 3 13 10.1177/0748730406296749 17229920

[B11] Abrahamson EE Moore RY Suprachiasmatic nucleus in the mouse: retinal innervation, intrinsic organization and efferent projections Brain Res 2001 916 172 191 10.1016/S0006-8993(01)02890-6 11597605

[B12] Kawamoto K Nagano M Kanda F Chihara K Shigeyoshi Y Okamura H Two types of VIP neuronal components in rat suprachiasmatic nucleus J Neurosci Res 2003 74 852 857 10.1002/jnr.10751 14648589

[B13] Shigeyoshi Y Taguchi K Yamamoto S Takekida S Yan L Tei H Moriya T Shibata S Loros JJ Dunlap JC Okamura H Light-induced resetting of a mammalian circadian clock is associated with rapid induction of the mPer1 transcript Cell 1997 7 1043 1053 10.1016/S0092-8674(00)80494-8 9428526

[B14] Watanabe K Vanecek J Yamaoka S In vitro entrainment of the circadian rhythm of vasopressin-releasing cells in suprachiasmatic nucleus by vasoactive intestinal polypeptide Brain Res 2000 877 361 366 10.1016/S0006-8993(00)02724-4 10986351

[B15] Hannibal J Fahrenkrug J Circadian rhythm regulation: a central role for the neuropeptide vasoactive intestinal polypeptide Am J Physiol Regul Integr Comp Physiol 2003 285 R935 R936 14557231 10.1152/ajpregu.00447.2003

[B16] Colwell CS Michel S Itri J Rodriguez W Tam J Lelievre V Hu Z Liu X Waschek JA Disrupted circadian rhythms in VIP-and PHI-deficient mice Am J Physiol Regul Integr Comp Physiol 2003 285 R939 R949 12855416 10.1152/ajpregu.00200.2003

[B17] Image J http://rsb.info.nih.gov/ij/

[B18] Colwell CS Foster RG Photic regulation of Fos-like immunoreactivity in the suprachiasmatic nucleus of the mouse J Comp Neurol 1992 324 135 142 10.1002/cne.903240202 1430326

[B19] Rea MA Michel AM Lutton LM Is fos expression necessary and sufficient to mediate light-induced phase advances of the suprachiasmatic circadian oscillator? J Biol Rhythms 1993 8 Suppl S59 S64 8274763

[B20] Ruby NF Brennan TJ Xie X Cao V Franken P Heller HC O'Hara BF Role of melanopsin in circadian responses to light Science 2002 298 2211 2213 10.1126/science.1076701 12481140

[B21] Shen S Spratt C Sheward WJ Kallo I West K Morrison CF Coen CW Marston HM Harmar AJ Overexpression of the human VPAC2 receptor in the suprachiasmatic nucleus alters the circadian phenotype of mice Proc Natl Acad Sci USA 2000 97 11575 11580 10.1073/pnas.97.21.11575 11027354 PMC17242

[B22] Aton SJ Colwell CS Harmar AJ Waschek J Herzog ED Vasoactive intestinal polypeptide mediates circadian rhythmicity and synchrony in mammalian clock neurons Nat Neurosci 2005 8 476 483 15750589 10.1038/nn1419 PMC1628303

[B23] Brown TM Colwell CS Waschek JA Piggins HD Disrupted neuronal activity rhythms in the suprachiasmatic nuclei of vasoactive intestinal polypeptide-deficient mice J Neurophysiol 2007 97 2553 2558 10.1152/jn.01206.2006 17151217 PMC2570696

[B24] Aton SJ Huettner JE Straume M Herzog ED GABA and Gi/o differentially control circadian rhythms and synchrony in clock neurons Proc Natl Acad Sci USA 2006 103 19188 19193 10.1073/pnas.0607466103 17138670 PMC1748197

[B25] Maywood ES Reddy AB Wong GK O'Neill JS O'Brien JA McMahon DG Harmar AJ Okamura H Hastings MH Synchronization and maintenance of timekeeping in suprachiasmatic circadian clock cells by neuropeptidergic signaling Curr Biol 2006 16 599 605 10.1016/j.cub.2006.02.023 16546085

[B26] Piggins HD Antle MC Rusak B Neuropeptides phase shift the mammalian circadian pacemaker J Neurosci 1995 15 5612 5622 7643205 10.1523/JNEUROSCI.15-08-05612.1995 PMC6577626

[B27] Harmar AJ An essential role for peptidergic signalling in the control of circadian rhythms in the suprachiasmatic nuclei J Neuroendocrinol 2003 15 335 338 10.1046/j.1365-2826.2003.01005.x 12622830

[B28] Liu C Reppert SM GABA synchronizes clock cells within the suprachiasmatic circadian clock Neuron 2000 1 123 128 10.1016/S0896-6273(00)80876-4 10707977

[B29] Gillespie CF Mintz EM Marvel CL Huhman KL Albers HE GABA(A) and GABA(B) agonists and antagonists alter the phase-shifting effects of light when microinjected into the suprachiasmatic region Brain Res 1997 759 181 189 10.1016/S0006-8993(97)00235-7 9221935

[B30] Castel M Morris JF Morphological heterogeneity of the GABAergic network in the suprachiasmatic nucleus, the brain's circadian pacemaker J Anat 2000 196 1 13 10.1046/j.1469-7580.2000.19610001.x 10697283 PMC1468035

[B31] Itri J Colwell CS Regulation of inhibitory synaptic transmission by vasoactive intestinal peptide (VIP) in the mouse suprachiasmatic nucleus J Neurophysiol 2003 3 1589 1597 10.1152/jn.00332.2003 12966176

[B32] Moore RY Speh JC GABA is the principal neurotransmitter of the circadian system Neurosci Lett 1993 150 112 116 10.1016/0304-3940(93)90120-A 8097023

[B33] Lamb AH Motoneuron death in the embryo CRC Crit Rev Clin Neurobiol 1984 1 141 179 6100836

[B34] Oppenheim RW Cell death during development of the nervous system Annu Rev Neurosci 1991 14 453 501 10.1146/annurev.ne.14.030191.002321 2031577

[B35] Linden R Perry VH Ganglion cell death within the developing retina: a regulatory role for retinal dendrites? Neuroscience 1982 7 2813 2827 10.1016/0306-4522(82)90104-X 7155355

[B36] Wright LL Smolen AJ The role of neuron death in the development of the gender difference in the number of neurons in the rat superior cervical ganglion Int J Dev Neurosci 1987 5 305 311 10.1016/0736-5748(87)90005-0 3503506

[B37] Foster RG Provencio I Hudson D Fiske S De Grip W Menaker M Circadian photoreception in the retinally degenerate mouse (rd/rd) J Comp Physiol 1991 169 39 50 10.1007/BF00198171 1941717

[B38] Tosini G Aguzzi J Bullock NM Liu C Kasamatsu M Effect of photoreceptor degeneration on circadian photoreception and free-running period in the Royal College of Surgeons rat Brain Res 1148 76 82 10.1016/j.brainres.2007.02.055 17382912 PMC1939936

